# A*β*1–40 Oligomers Trigger Neutrophil Extracellular Trap Formation through TLR4- and NADPH Oxidase-Dependent Pathways in Age-Related Macular Degeneration

**DOI:** 10.1155/2022/6489923

**Published:** 2022-06-18

**Authors:** Jinquan Chen, Long Zhao, Xuanheng Ding, Yan Wen, Lingda Wang, Qinxin Shu, Wenxi Xie, Yanyao Liu, H. Peng

**Affiliations:** ^1^Department of Ophthalmology, The First Affiliated Hospital of Chongqing Medical University, Chongqing, China; ^2^Chongqing Key Laboratory of Ophthalmology, Chongqing Eye Institute, Chongqing, China

## Abstract

Neutrophils participate in the advancement of the human innate immune system and respond to perceived endogenous and exogenous threats. As a response mechanism, neutrophil extracellular traps (NETs) form near pathogens and surrounding tissues during an immune response. Drusen is an important marker of Age-Related Macular Degeneration (AMD) and plays an important role in the course of AMD. A*β*1-40 is the main component of drusen. However, the relationship between NETs and AMD or A*β*1-40 is unclear. Here, we found elevated levels of NETs in the serum of AMD patients and elevated levels in the serum of mouse models. We also observed the accumulation of neutrophils in the mouse retina. In addition, the production of NETs was inhibited by PAD4 inhibitors, which can alleviate chronic inflammation. Moreover, we confirmed that A*β*1-40 can induce NETs formation via the Toll-like receptor 4 (TLR4) and neutrophil NADPH oxidase (NOX) pathways. Our study confirmed that the formation of NETs is induced by A*β*1–40, and the results suggest that NETs may play a vital role in AMD pathogenesis.

## 1. Introduction

Age-related macular degeneration (AMD) is one of the major causes of irreversible visual loss in the elderly [1]. With the ageing of populations in industrialized countries, the incidence of AMD is increasing. One early feature of AMD is an extracellular fragment called drusen, which is found mainly between the retinal pigment epithelium (RPE) and Bruch's (BrM) membrane. As the disease progresses, these drusen expand and contact, resulting in two distinct late disease manifestations. One is geographic atrophy (GA), characterized by irreversible damage to retinal cells. One is osmotic AMD, characterized by choroidal neovascularization (CNV) [2]. Both conditions can lead to reduced vision and quality of life.

A*β* is a major constituent of drusen and induces pathological processes [3]. A study showed that A*β*1–40 induces the release of inflammatory factors and activates oxidative stress in the retina [4, 5]. A*β*1–40 also has been found to cooperate with neutrophils to trigger cognitive damage in Alzheimer's disease (AD) [6]. Recently, intravitreal injection of A*β*1-40 has replaced subretinal injection and has been used to establish AMD mouse models [7]. The presence of A*β*1-40 triggers a local inflammatory response, thereby activating the immune system [8]. In addition, A*β*1-40 activates multiple immune pathways. The expression of NADPH oxidase (NOX) and Toll-like receptors (TLRs) has been found to rise after the intravitreal injection of A*β*1-40 [9].

Neutrophils are highly differentiated immune cells thought to be the main players in chronic inflammation [10]. In the early immune response, neutrophils participate in inflammation through multiple mechanisms, including phagocytosis, degranulation, the induction of inflammation, and the organization of neutrophil extracellular traps (NETs) [11]. Therefore, neutrophils may also aggravate the inflammatory response and cause tissue damage [12].

NETosis, which is a form of cell death specific to neutrophils, is characterized by the release of NETs into the extracellular space to defend against pathogens [13]. NETs are composed of DNA and various proteins, including four kinds of histones, neutrophil elastase (NE), and myeloperoxidase (MPO) [14]. NETosis can be induced by NOX and TLRs [15, 16]. Additionally, the activation of neutrophils and the release of NETs are triggered by specific receptors of biomolecules, such as TLRs [17].

The production of NETs contributes to the clearance of pathogens. Excessive NETs production may cause autogenic immune diseases such as systemic lupus erythematosus [18, 19]. Previous studies have found neutrophil enrichment in human subjects with early AMD and a model of AMD [20]. Upon reaching tissues, neutrophils begin to release NETs, which are involved in inflammation and ultimately lead to pathological sequelae [21]. Additionally, recent studies have found that NETs are present in a variety of eye diseases, such as uveitis and dry eye disease [22]. Furthermore, patients with diabetic retinopathy have NETs in their vitreous bodies, and anti-VEGF treatment can significantly degrade NETs levels in the vitreous body [23]. Anti-VEGF therapy is also a main treatment option for patients with AMD [24]. Thus, we speculate that anti-VEGF treatment delays the pathophysiological progression of AMD by reducing the production of NETs.

Here, we hypothesized that A*β*1–40 may trigger the production of NETs by stimulating TLR4 and NOX. We studied AMD patients and identified the pathway by which A*β* triggers the production of NETs. We explored the mechanism underlying the occurrence and development of AMD, providing a theoretical basis for the treatment of AMD.

## 2. Materials and Methods

### 2.1. Human Blood Samples

This work was authorized by Chongqing Medical University's Animal and Human Experimentation Ethics Committee (Approval number: 2021-712). All human subjects provided informed consent to participate in the study. All blood sample collection procedures conformed to the Declaration of Helsinki and the principles of the ARVO Statement on Human Subjects. Peripheral venous blood samples were gathered from 20 healthy controls (HCs) aged 60 years and 21 AMD patients not undergoing any therapy. The AMD patients were diagnosed by professional ophthalmologists by optical coherence tomography and optical coherence angiography based on the revised criteria. To avoid potential baseline activation, donors with cardiovascular diseases, autoimmune diseases, haematological diseases, active infections, and high neutrophil counts were excluded. The control subjects were age- and sex-matched with the AMD patients. Peripheral blood was collected from ten healthy donors for in vitro experiments.

### 2.2. Isolation and Identification of Primary Human Neutrophils

Peripheral venous blood was extracted from all blood donors and collected in K2-EDTA blood collection tubes (BD, CA, USA). Serum was acquired by centrifugation (300 × *g*, 10 min, 21°C) and stored at -80°C until use. Primary human neutrophils were obtained with a human peripheral neutrophil separation kit (TBD, Beijing, China), and red blood cells were removed with red blood cell lysis buffer. Subsequently, the purity of the neutrophils (>98%) was determined by analysis of CD11b (BD, CA, USA) and CD16 (BD, CA, USA) expression using flow cytometry, and the neutrophils were tallied using a cell counter. The cell viability was >95% and was determined via trypan blue dye.

### 2.3. Animal Model and Treatment

Two-month-old C57BL/6 mice (male) were obtained from the Animal Care Committee of Chongqing Medical University. All mice were reared and housed in sterilized enclosures with a 12 h light cycle. All experimental methods were approved by the Ethics Committee of Chongqing Medical University. The mice were anaesthetized by the intraperitoneal injection of 1.5% sodium pentobarbital (5 *μ*l/g), and A*β*1-40 (1.5 *μ*g/2 *μ*l) was injected into the vitreous under a microscope using a microsyringe; PBS (2 *μ*l) was injected into vitreous as control animals. Moreover, GSK484 (TargetMol, Boston, USA), a PAD4 inhibitor (4 mg/kg daily), was injected intraperitoneally into mice in the inhibition group. The mice were sacrificed 2 and 4 days after intravitreal injection, and the eyeballs and peripheral blood were collected and stored at -80°C.

### 2.4. Quantitative RT–PCR

The retinas were isolated, and total mRNA was extracted from each sample by TRIzol (Carlsbad, CA, USA). Quantitative PCR was performed using an Applied Biosystems 7500 Fast Real-Time PCR System (Foster City, CA, USA). The following PCR primers were used: TNF-*α*, 5′-GCCTCTTCTCATTCCTGCTT-3′ and 5′-CTCCTCCACTTGGTGGTTTG-3′; IL-6, 5′-ACCACGGCCTTCCCTACTTC-3′ and 5′-AGATTGTTTTCTGCAAGTGCATCA-3′; IL-1*β*, 5′-TCCTTGTGCAAGTGTCTGAAGC-3′ and 5′-ATGAGTGATACTGCCTGCCTGA-3′; Ly6G, 5′-TCAGCCTGGTTCAGTCCTTC-3′ and 5′-AGGAGTGGGGTGCCTATACA-3′; and *β*-actin, 5′-GACAGG ATGCAGAAGGAGATTACT-3′ and 5′TGATCCACATCTGCTGGAAGGT-3′. All specific primer sequences were designed by Sangon Biotech (Shanghai, China). All samples were analyzed in triplicate with normalization to the *β*-actin expression level in the sham group.

### 2.5. Histological Analysis

Mouse eyes were fixed with PFA (Solarbio, Beijing, China) for 24 h, embedded in paraffin, sectioned into 5 *μ*m slices, and stained with haematoxylin and eosin (H&E). A fluorescence microscope was used to observe and image the paraffin sections.

### 2.6. Formation of A*β*1–40 Oligomers

The oligomer A*β*1-40 peptide (Sigma, St. Louis, USA) was prepared according to the manufacturer's instructions. In short, the freeze-dried A*β*1-40 peptide was dissolved in deionized distilled water (6 *μ*g/*μ*l). Before incubating at 37°C for 4 days, the A*β*1-40 oligomer was diluted with phosphate-buffered saline to a concentration of 1.5 *μ*g/*μ*l and stored at -20°C until use. A*β*40-1 peptide (Sigma, St. Louis, USA) was used as a negative control for A*β*1-40, and A*β*40-1 was handled in the same way as A*β*1-40.

### 2.7. Neutrophil Stimulation

A total of 1 × 10^6^ neutrophils were seeded into each well of a 6-well plate in 1 ml of low-glucose DMEM (Gibco, Grand Island, USA) containing A*β*1–40 (0.1 *μ*M, 1 *μ*M, or 10 *μ*M), 200 nM PMA (MCE, Columbia, USA), and 10 *μ*g/ml LPS (TargetMol, Boston, USA) and incubated at 37°C and 5% CO_2_ for 4 h. For the use of inhibitors, neutrophils were treated with 5 *μ*M diphenyleneiodonium chloride (DPI) (MCE, Columbia, USA) or 1 *μ*M TAK-242 (TargetMol, Boston, USA) for 30 min before inducing NETosis. Neutrophils were treated with LPS for 30 min followed by the addition of TAK-242 and A*β*1-40.

### 2.8. Quantification of Extracellular DNA Levels

A Quant-it Pico-Green dsDNA Assay Kit (Sigma, St. Louis, USA) was used to measure extracellular DNA levels in serum and medium to evaluate NETs formation. Pico-Green is a fluorescent dye that labels extracellular DNA but does not stain living cells. A microplate reader (Thermo, Waltham, USA) was used to measure the fluorescence signal intensity at an excitation wavelength of 485 nm and an emission wavelength of 535 nm. Each sample was measured in duplicate.

### 2.9. Superoxide Assay

2′,7′-Dichlorofluorescein diacetate was used to measure the generation of ROS. Isolated neutrophils were resuspended in 1 ml of low-glucose medium, and 10 *μ*M DCFH-DA (Beyotime Biotechnology, Shanghai, China) was added. After 20 min, the cells were washed with low-glucose medium three times and then stimulated with A*β*1–40, A*β*40–1, or PMA. Finally, the fluorescence intensity of DCFH-DA was measured at an excitation wavelength of 488 nm and an emission wavelength of 525 nm with a microplate reader (Thermo, Waltham, USA).

### 2.10. ELISA

NE levels in human peripheral blood and medium were measured using enzyme-linked immunosorbent assay kits (JYM, Xian, China) according to the manufacturer's instructions.

### 2.11. Western Blotting

Neutrophil and total mouse retina proteins were extracted using RIPA buffer. A BCA kit was used to measure the protein concentration. The proteins were separated on 12% sodium dodecyl sulfate–polyacrylamide gel electrophoresis and then transferred to polyvinylidene fluoride membranes, which were blocked with blocking buffer for 15 min. The polyvinylidene fluoride membranes were incubated with primary antibodies against *β*-actin, JNK, p-JNK, ERK, p-ERK, NOX2, NOX4, P38, p-P38, and TLR4 at 4°C overnight, followed by incubation with the secondary antibody for 1.5 h and subsequent washing with PBS three times. The above antibodies were purchased from Affinity (Jiangsu, China).

### 2.12. Immunofluorescence

To observe the production of NETs, neutrophils were seeded into 24-well plates precoated with polylysine and then treated as described above for 4 h at 37°C. DAPI was used to stain DNA. Neutrophil-specific enzymes in the NETs structure were labelled with primary antibodies against H3-cit (Abcam, Cambridge, UK) and MPO (Abcam, Cambridge, UK), and the primary antibodies were detected with a secondary antibody. The localization and structure of NETs were determined by using an Olympus laser scanning confocal microscope (Olympus, Tokyo, Japan).

### 2.13. Statistical Analysis

All data are represented as the mean ± SEM and were derived from at least 3 independent replicates. *T*-test analysis, one-way ANOVA, Mann–Whitney test, and Chi-square test were performed using GraphPad Prism 8.0 software and SPSS version 2.0. *P* < 0.05 was a significant difference.

## 3. Results

### 3.1. Elevated Serum Levels of NETs in AMD Patients and Mouse Models

**Figure 1:**
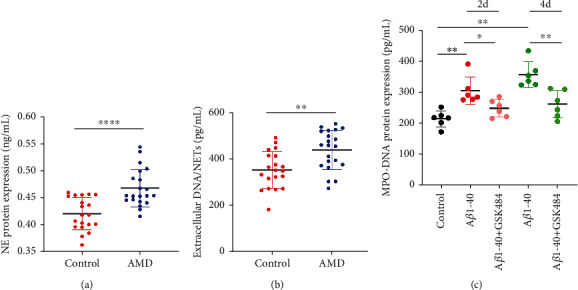
Circulating NE production and extracellular DNA/NETs are elevated in sera from individuals with AMD. (a) Circulating NE levels were measured in the sera of HCs and individuals with AMD. (b) Extracellular DNA/NETs levels were increased in the sera of individuals with AMD compared to the sera of HCs. *n*_HCs_ = 20; *n*_AMD_ = 21. (c) Enhanced NETs production in the sera of mice injected with A*β*1-40 compared to control animals and reduced NETs production in sera after GSK484 intraperitoneal injection. ^∗^*P* < 0.05, ^∗∗^*P* < 0.01, ^∗∗∗∗^*P* < 0.0001.

The levels of extracellular DNA and NE, which are components of NETs, are often used to quantify NETs contents. To evaluate the production of NETs, we measured extracellular DNA and NE levels in the sera of AMD patients (Figures 1(a) and 1(b)). In the mouse model, MPO-DNA levels were increased in the sera and decreased after intraperitoneal injection of GSK484 (Figure 1(c)). These data show that serum levels of NETs in AMD patients are higher than those in healthy people of the same age group. The NETs content in AMD model mice was higher than that in control mice, and GSK-484 effectively inhibited the production of NETs.

### 3.2. Retinal Inflammation Is Reduced after Inhibiting NETs Formation In Vivo

**Figure 2:**
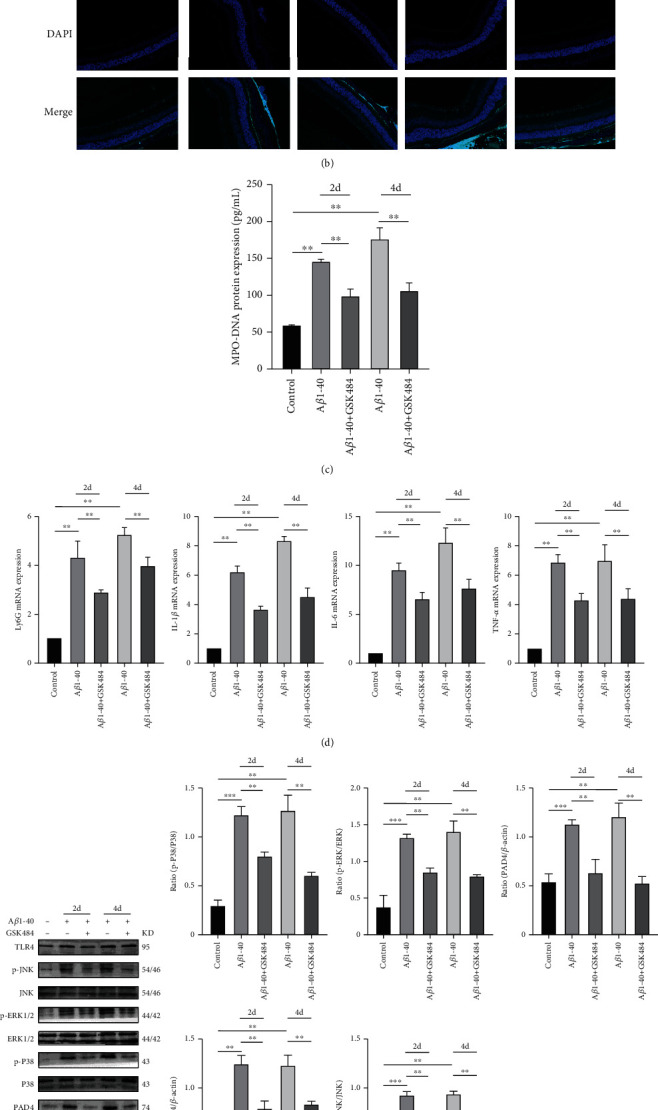
GSK484 alleviates retinal inflammation induced by A*β*1-40. (a) H&E staining of retinopathy indicates pathology photomicrographs. Scale bars = 50 *μ*m. (b) Immunofluorescence analysis of retinal neutrophils labelled with the neutrophil marker Ly6G. Scale bars = 100 *μ*m. (c) Detection of MPO-DNA in mouse vitreous. (d) The expression of Ly6G, IL-6, IL-1*β*, and TNF-*α* in the retinas was quantified by qRT–PCR. (e) Western blot analysis of H3-cit, PAD4, TLR4, JNK, p-JNK, ERK, p-ERK, P38, and p-P38 expression in retinas. ^∗∗^*P* < 0.01.

To further study the relationship between NETs and chronic inflammation of the retina, we verified the deposition of neutrophils in the retina. In the mouse model, H&E staining revealed that compared with the control group, the retinal structure of the A*β*1-40 group was disordered, and the retinal structure recovered after treatment with GSK-484 (Figure 2(a)). Then, we used immunofluorescence to localize neutrophils in the mouse retina, and we found neutrophil infiltration in the retinas of both AMD and control mice (Figure 2(b)). To determine whether NETs were elevated in the AMD mice, we collected vitreous from the mice and measured the amount of MPO-DNA in the vitreous. The content of MPO-DNA in the vitreous of AMD mice was higher than that in the control group. The MPO-DNA content decreased after GSK-484 treatment (Figure 2(c)). In addition, A*β*1-40 elevated retinal PAD4 expression in mouse retinas, while GSK484 inhibited this effect (Figure 2(e)). To further determine the role of NETs, we examined the expression of Ly6G and inflammatory factors in the mouse retina. Compared with the control group, Ly6G, IL-1*Β*, IL-6, and TNF-*α* expression increased in the retinas of AMD mice, while GSK-484 effectively inhibited this increase (Figure 2(d)). Western blotting revealed that A*β*1-40 can activate TLR4 and the phosphorylation of JNK, ERK, and P38. After suppressing NETs, this effect was reduced (Figure 2(e)). These results suggest that NETs may aggravate A*β*1-40-induced retinal inflammation in a mouse model by TLR4/MAPK pathway.

### 3.3. Neutrophils from AMD Patients Easily Produce NETs In Vitro

**Figure 3:**
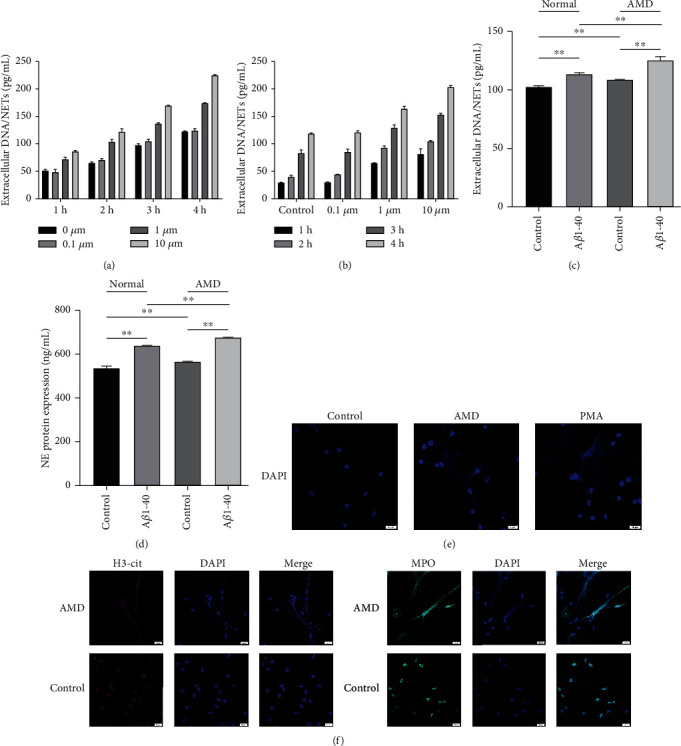
Release of NETs from neutrophils from donors with AMD. (a and b) The release of NETs was quantified every hour from 1 h to 4 h and from 0 *μ*M to 10 *μ*M. (c and d) Measurements of extracellular DNA and NE levels indicated the presence of NETs. Scale bars = 20 *μ*m. (e) Human neutrophils were isolated from the peripheral blood of individuals with AMD and HCs and incubated at 37°C for 4 h in the absence of any stimulation. PMA was considered the positive group. (f) Immunofluorescence analysis of H3-Cit and MPO and their colocalization with extracellular DNA. Scale bars = 20 *μ*m. ^∗∗^*P* < 0.01.

To further demonstrate the influence of A*β*1–40 oligomers on NETs formation, we identified the effects in vitro. We isolated neutrophils using healthy human peripheral blood and cultured them in DMEM at different concentrations of A*β*1-40 (0, 0.1, 1, and 10 *μ*M) for different times (60, 120, 180, and 240 min) to determine NETs formation and stimulation conditions. The results showed that the 10 *μ*M concentration of A*β*1-40 had the strongest effect at 4 h (Figures 3(a) and 3(b)). We then assessed the ability of neutrophils from AMD patients to produce NETs. The results showed that neutrophils from AMD patients induced more NETs production than neutrophils from control subjects in vitro (Figures 3(c) and 3(d)).

NETs are DNA-based structures that contain a variety of proteins. After isolating and purifying neutrophils, we observed the structure of NETs using immunofluorescence. PMA was used as a positive control. In in vitro, neutrophils from AMD patients produced NETs compared to the healthy group and were similar to the positive control (Figure 3(e)). To confirm NETosis, the NETs components H3-cit and MPO were examined (Figure 3(f)). All of the collected data showed that neutrophils from AMD patients are more likely to produce NETs than those from healthy individuals.

### 3.4. A*β*1–40 Oligomers Induce NETs Formation In Vitro

**Figure 4:**
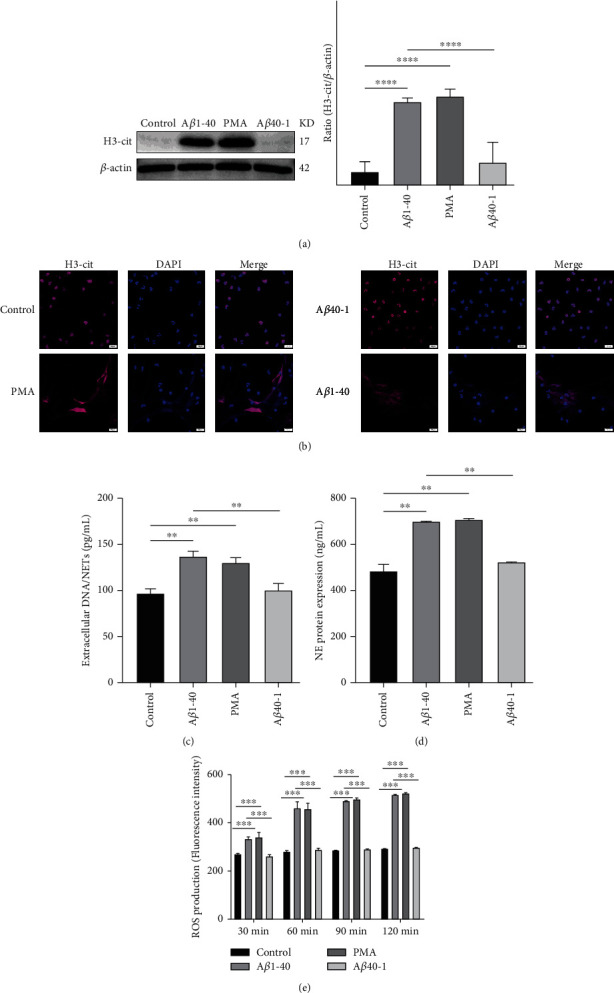
Extracellular A*β*1–40 oligomers induce NETs formation. (a) Western blot analysis of H3-cit expression in neutrophils following the treatment described above. (b) Neutrophils were incubated with PMA (200 nM), A*β*1–40 oligomers (10 *μ*M), A*β*40–1 (10 *μ*M), or the control for 4 h at 37°C. DAPI was used to label extracellular DNA. H3-cit was immunolabelled with an antibody. Scale bars = 20 *μ*m. (c and d) Measurements of extracellular DNA and NE levels indicated the presence of NETs. (e) ROS production induced by treatment at different concentrations, as quantified by preloading with the DCFH-DA probe and measured with a microplate reader. ^∗∗^*P* < 0.01, ^∗∗∗^*P* < 0.001, ^∗∗∗∗^*P* < 0.0001.

A*β* is a major component of drusen that induces chronic inflammation in the retina of AMD patients. To determine the formation of NETs induced by A*β*1-40, we isolated neutrophils from human peripheral blood. After treatment with A*β*1-40, we used immunofluorescence to observe the structure of DAPI-stained DNA and its attached H3-cit, and we observed a net-like structure similar to that seen after treatment with the typical NETs stimulator PMA (Figure 4(a)). Prior to the release of NETs into the extracellular environment, histone H3 undergoes citrullination mediated by the enzyme PAD4; thus, we quantified H3-cit using Western blotting. The results showed elevated levels of citrullination of histone H3 in the A*β*1-40 group compared to the control group, which was consistent with the results of the PMA group (Figure 4(b)). Additionally, the expression of NE protein and dsDNA was elevated in the A*β*1-40 group (Figures 4(c) and 4(d)). To investigate whether A*β*1-40-induced NETs formation is ROS dependent, we used a DCHF-DA probe loaded into neutrophils. After A*β*1-40 stimulation, ROS levels were elevated in neutrophils (Figure 4(e)).

### 3.5. A*β*1–40 Oligomer-Induced NETs Formation Requires NOX In Vitro

**Figure 5:**
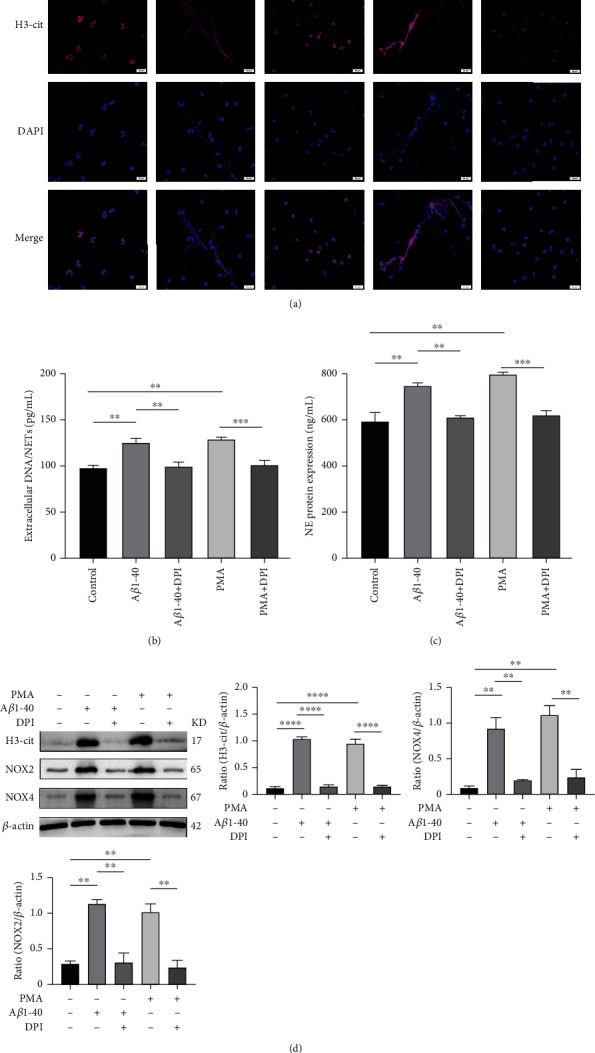
A*β*1–40 oligomers induce NETs formation via NOX. (a) Neutrophils were incubated with PMA (200 nM), A*β*1–40 oligomers (10 *μ*M), or the control for 4 h at 37°C. DAPI was used to label extracellular DNA. H3-cit was immunolabelled with an antibody in the absence or presence of the NOX inhibitor DPI (10 *μ*M). Scale bars = 20 *μ*m (b). (c) Measurements of extracellular DNA and NE levels indicated the presence of NETs after the treatment described above. (d) Western blot analysis of H3-cit, NOX2, and NOX4 expression in neutrophils following the treatment described above. ^∗∗^*P* < 0.01, ^∗∗∗^*P* < 0.001, ^∗∗∗∗^*P* < 0.0001.

Previous studies have shown that there are two pathways for the formation of NETs: NOX-dependent and NOX-independent pathways. We isolated neutrophils from human peripheral blood. In in vitro, prior to the induction of NETosis using A*β*1-40, neutrophils were pretreated with DPI for 30 min. As shown by immunofluorescence, we found that DPI inhibited both A*β*1-40- and PMA-induced NETs formations (Figure 5(a)). In addition, the expression of NE proteins and dsDNA decreased (Figures 5(b) and 5(c)), while citrullination of histone H3, NOX2, and NOX4 was detected by Western blot and decreased with DPI treatment (Figure 5(d)). This finding is consistent with the results of other studies showing that DPI can inhibit the formation of NETs. These results suggest that A*β*1-40 may induce the formation of NETs through the NADPH pathway.

### 3.6. TLR4 Inhibitors Effectively Reverse the Formation of NETs Induced by A*β*1-40 Oligomers In Vitro

**Figure 6:**
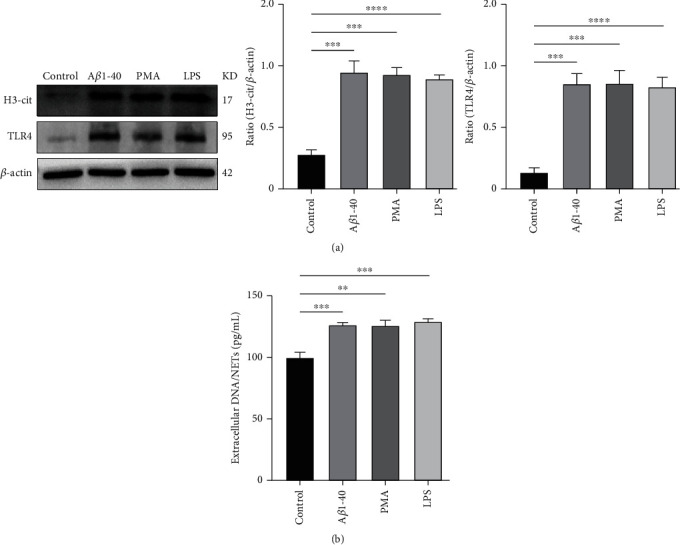
LPS induces NETs formation. (a) Western blot analysis of H3-cit expression in neutrophils treated with PMA (200 nM), A*β*1–40 oligomers (10 *μ*M), LPS (10 mg/ml), or the control. (b) Measurements of extracellular DNA levels indicated the presence of NETs after the treatment described above. ^∗∗^*P* < 0.01, ^∗∗∗^*P* < 0.001, ^∗∗∗∗^*P* < 0.0001.

Neutrophils express a variety of TLRs, and their activation has been reported in previous studies to be effective in inducing the formation of NETs, such as LPS, bacteria, and viruses. Previous studies have shown that activating TLR4 can effectively induce NETs formation. We treated neutrophils with A*β*1-40, LPS, and PMA and found that citrullination of histone H3 expression increased and TLR4 expression increased (Figure 6(a)). In addition, the content of dsDNA in the medium increased (Figure 6(b)).

**Figure 7:**
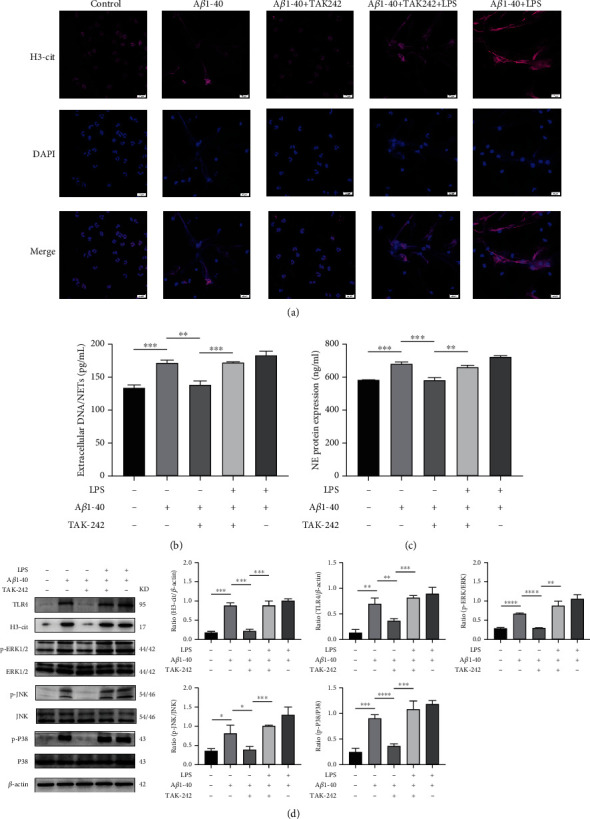
TLR4 mediates NETs formation through A*β*1–40 oligomers. (a) Neutrophils were incubated with PMA (200 nM), A*β*1–40 oligomers (10 *μ*M), LPS (10 mg/ml), or the control for 4 h at 37°C. DAPI was used to label extracellular DNA. H3-cit was immunolabelled in the absence or presence of the TLR4 inhibitor TAK-242 (10 *μ*M) or TLR4 agonist LPS (10 mg/ml). Scale bars = 20 *μ*m (b). (c) Measurements of extracellular DNA and NE levels indicated the presence of NETs after the treatment described above. (d) Western blot analysis of H3-cit, TLR4, JNK, p-JNK, ERK, p-ERK, P38, and p-P38 expression in neutrophils following the treatment described above. ^∗^*P* < 0.05, ^∗∗^*P* < 0.01, ^∗∗∗^*P* < 0.001, ^∗∗∗∗^*P* < 0.0001.

We investigated whether A*β*1-40 induces NETosis through TLR4 activation. We isolated neutrophils from human peripheral blood. Prior to the induction of NETosis, we treated neutrophils with TAK-242 for 30 min. By immunofluorescence, we found that NETs formation could not be observed in neutrophils after TLR4 inhibition; LPS reversed this effect, and LPS had no significant effect on neutrophils in the A*β*1-40 group. (Figure 7(a)). Changes in NE protein and dsDNA were consistent with the immunofluorescence results (Figures 7(b) and 7(c)).

To further determine whether A*β*1-40-induced NETosis was attributed to TLR4 activation, we examined TLR4 protein expression using WB and showed that A*β*1-40 induced a rise in TLR4 expression and thus induced the citrullination of histone H3. TAK-242 inhibited the rise in TLR4 and reduced citrullination of histone H3. LPS reversed this change (Figure 7(d)). Subsequently, we examined the phosphorylation of JNK, P38, and ERK on the MAPK pathway. The results showed that A*β*1-40 could induce the phosphorylation of JNK, P38, and ERK, and TAK-242 could effectively inhibit the phosphorylation of these proteins (Figure 7(d)). These results suggest that A*β*1-40 can induce NETosis by activating TLR4 to induce the MAPK pathway.

## 4. Discussion

Previous studies have found increased retinal neutrophils in AMD patients and AMD mice [25]. Our study further confirmed the increased expression of NETs in the serum of AMD patients and AMD mice. However, the mechanisms by which neutrophils form NETs in patients with AMD and whether NETs are involved in the pathogenesis of AMD are unclear. Our study demonstrated that NETs are possibly induced by A*β*1-40 via the TLR4/NADPH pathway.

Neutrophils are a type of innate immune cell and a source of elastase that is widely involved in aseptic inflammation processes [15]. When subjected to an immune response, neutrophils can remove pathogens by releasing net-like structures (i.e., NETs). NETs are composed of extracellular chromatin, nucleoprotein, and serine proteases [26]. ELISA and Pico-Green kit can be used to determine the components of NETs, such as NE, MPO-DNA, and dsDNA. We found that the expression levels of NE and dsDNA were increased in the serum of patients with AMD, and the expression of the MPO-DNA complex was increased in the peripheral blood of the A*β*1-40-injected mouse model. The formation of NETs depends on PAD4 enzyme activation. When PAD4 is inhibited, NETs formation is also inhibited [27]. Our results show that increased levels of NETs in AMD patients and AMD model mice and inhibition of PAD4 can effectively inhibit NETs formation in mice.

Based on the above serological results, we conducted further studies to investigate the role of NETs in the pathogenesis of AMD. Previous studies have shown that intravitreal injection of A*β*1-40 causes retinal inflammation. This pathological change is similar to the response observed in the early stages of AMD [7]. Our research revealed that after A*β*1-40 was injected into the vitreous body, the structure of the mouse retina was disordered, and neutrophils migrated to the retina. After inhibiting NETs formation, the structure of the mouse retina was restored, and retinal inflammation was reduced. Our data also reveal elevated levels of NETs in the vitreous of AMD mice. These results are consistent with those obtained in previous studies on NETs, indicating that NETs may promote the development of inflammation [28]. These results indicate that inhibiting NETs formation can effectively reduce A*β*1-40-induced retinal inflammation. NETs may play an important role in AMD progression. In addition, A*β*1-40 can activate the TLR4 and MAPK pathways. Previous studies have found that activation of these pathways induces NETosis.

To further confirm whether the formation of NETs is related to A*β*1-40, we isolated neutrophils from AMD patients and healthy people as study samples. We found that A*β*1-40 can induce NETs formation in a time-dependent manner. To test the ability of neutrophils to form NETs, in this study, we found an increase in spontaneously formed neutrophil NETs in AMD patients compared to healthy controls and more neutrophil NETs in AMD patients compared to healthy controls. It may depend on the state of the neutrophils in the patient. We also found that A*β*1-40 can induce similar net-like structures in vitro, which is consistent with that induced by the classical NETs inducer PMA [29] but not by A*β*40–1, which was used as a negative control for A*β*1–40 [30].

Previous studies have shown that NETs formation can be initiated through NOX-dependent or NOX-independent pathways after stimulation [16]. According to previous studies, oxidative stress is triggered by A*β*1–40 oligomers [31]. Therefore, the effect of A*β*1-40 on oxidative stress in neutrophils should be studied. Our data show that ROS levels in neutrophils increased significantly during NETosis induction by A*β*1-40, and ROS production was the highest after 1 h of A*β*1–40 oligomer stimulation, after which the production of ROS began to decrease. A*β*1–40 oligomers activated the NADPH pathway, and NETosis was inhibited by DPI. A*β*1–40 oligomers induced NETs formation via a NOX-dependent pathway. These findings may provide a new target for the treatment of AMD.

Pattern recognition receptors in neutrophils can be activated by extracellular infectious or sterile threats. The activation of these receptors has different effects on cell survival, chemotaxis, and gene regulation. Previous studies have shown that various TLRs mediate the production of NETs [32]. Studies have found that the activation of the TLR4 and MAPK pathways plays an important role in the development of AMD. In addition, A*β*1–40 oligomers have been shown to participate in the development of AMD through the TLR4 pathway. In a follow-up experiment, A*β*1–40 oligomers were unable to induce NETs formation after treatment with a TLR4-specific inhibitor. LPS can restore the function of neutrophils after inhibition by TLR4-specific inhibitors. Furthermore, we investigated the role of TLR4-mediated protein kinase activation and MAPKs in A*β*1–40 oligomer-induced NETs formation. A*β*1–40 oligomers increased the phosphorylation of ERK, JNK, and p38 to induce NETs formation. These results confirm that A*β*1–40 can induce NETs formation via the TLR4 pathway.

Drusen is a crucial pathological feature of AMD. RPE cells, retinal cells, and retinal vascular endothelial cells are all affected by the extracellular inflammatory environment induced by drusen [33]. Our data illustrate that NETs formation was induced by A*β*1–40. The induction of NETs formation by A*β*1–40 was found to be related to the TLR4 and NOX pathways. This study reveals the mechanism of AMD and provides a theoretical basis for AMD treatment. However, it is unclear how NETs influence the development of AMD. Because the composition of NETs is relatively complex, their components play different roles in diseases, and these roles deserve further study.

## 5. Conclusion

Our results revealed that A*β*1-40 oligomers induced NETs production through the TLR4 and NOX pathways. Inhibiting the production of NETs effectively reduced the retinal inflammatory response. NETs may be a potential therapeutic target as well as a possible explanation for the occurrence and progression of AMD.

## Data Availability

The data of this study is included within the article. The data is available from the first author (chenj187@163.com) upon request.
